# Impact of Periodontal Host-Modulation Therapies on Oral–Gut Microbiome Axis in Periodontitis Patients with Hematological Diseases: A Narrative Review

**DOI:** 10.3390/life15121862

**Published:** 2025-12-04

**Authors:** Bianca Maria Messina, Alessandro Polizzi, Cristina Panuzzo, Antonio Belmonte, Angela Angjelova, Virginia Fuochi, Marco Annunziata, Gaetano Isola

**Affiliations:** 1 Unit of Periodontology, Department of General Surgery and Surgical-Medical Specialties, School of Dentistry, University of Catania, 95123 Catania, Italy; 2 International Research Center on Periodontal and Systemic Health “PerioHealth”, University of Catania, 95123 Catania, Italy; 3 Department of Clinical and Biological Sciences, University of Turin, 10124 Orbassano, Italy; 4 Department of Biomedical and Biotechnological Sciences, University of Catania, Via Santa Sofia 97, 95124 Catania, Italy; 5 Multidisciplinary Department of Medical-Surgical and Dental Specialties, University of Campania “Luigi Vanvitelli”, 81100 Naples, Italy

**Keywords:** anti-inflammatory agents, cancer immunotherapy, hematologic neoplasms, periodontitis, periodontal therapy, probiotics

## Abstract

Host-modulating therapies and oral microbiome-targeted approaches are emerging options in periodontal care and are especially relevant for patients undergoing immunotherapy for hematologic malignancies. Immune dysregulation induced by immune checkpoint inhibitors or CAR-T cell therapy may worsen periodontal inflammation and alter the composition and functions of the oral microbiota. Beyond these, other immunomodulatory treatments commonly employed in hematologic malignancies—including monoclonal antibodies (e.g., rituximab, daratumumab), immunomodulatory drugs (e.g., lenalidomide, thalidomide), cytokine-based therapies (e.g., interferon-α), and targeted small-molecule inhibitors (e.g., BTK inhibitors, JAK inhibitors) may also influence periodontal homeostasis and oral microbial ecology by altering neutrophil function, cytokine profiles, and mucosal immune surveillance. The oral microbiota is functionally connected with the intestinal microbial ecosystem through the oral–gut axis, by periodontal pathogens may colonize the gut and modulate systemic immune responses, with potential repercussions on the efficacy and safety of immunotherapy. This narrative review examines the mechanisms and clinical applicability of host-modulating therapies, including subantimicrobial-dose doxycycline, omega-3 fatty acids, and microbiome-targeted interventions, such as oral probiotics, prebiotics and other antimicrobials in patients treated with immunotherapy.

## 1. Introduction

Periodontitis is a chronic inflammatory condition that affects the structures supporting the teeth. Periodontitis results from dysbiosis of the oral microbiome and affects up to 70% adults aged 65 years and older [1]. Periodontitis is now understood as a positive-feedback loop between the oral microbiome and the host inflammatory response [2]. The accumulation of a bacterial biofilm (bacterial plaque) on the surface of the teeth can elicit a local immune response from the host and periodontal treatment could impact the overall burden on gingival microbiome [2].

**Figure 1:**
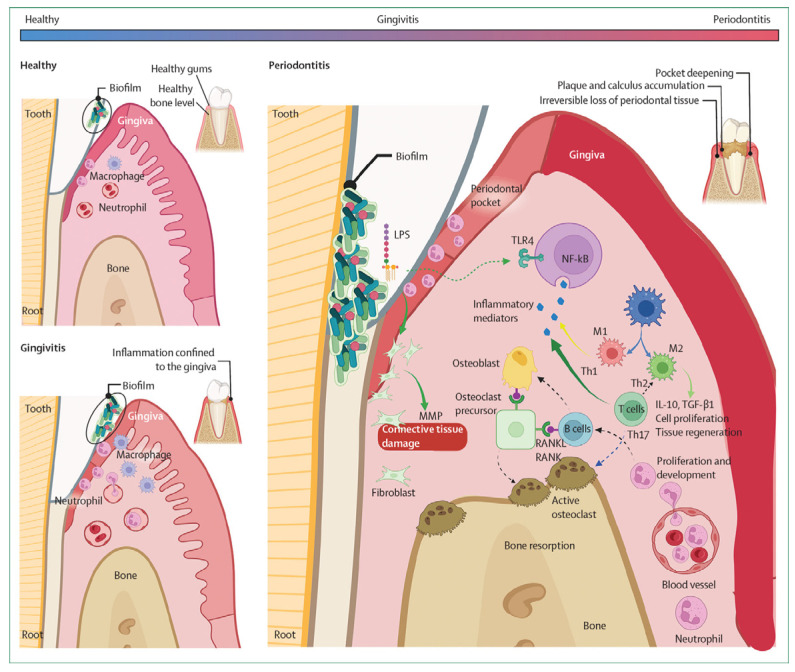
Pathological cascade of plaque-induced periodontal disease. The diagram depicts the sequential clinical, cellular, and molecular events that characterize the shift from periodontal homeostasis to tissue breakdown. Adapted from Muñoz-Carrillo et al. [3]. LPS = lipopolysaccharide; MMP = matrix metalloproteinase; Th = T helper cell. From [1], with permission.

Recent studies demonstrated that more 50 systemic inflammatory disorders and comorbidities are associated with periodontitis, many of which overlap with immunotherapy-associated toxicities [3] (Figure 1).

Immunotherapy has changed the treatment landscape for patients with cancer by improving overall survival and providing durable response rates, but it has toxicity profiles distinct from those of traditional cytotoxic and targeted therapies [4]. Over 50% of patients with cancer in high-income countries are estimated to be eligible for treatment with immune checkpoint blockade (ICB) drugs [5]. Immunotherapy prompts the host immune system to eliminate cancer cells, but it can also inadvertently activate immune cells to recognize and target self-antigens, producing autoimmune-related toxic effects. Acute toxicities are common, and chronic immune-related adverse events reportedly affect up to 40% of patients with cancer treated with immunotherapy [6]. The toxicity profile of adverse events attributed to immunotherapy overlaps with many of the systemic inflammatory conditions possibly associated with periodontitis, such as diabetes, rheumatoid arthritis, inflammatory bowel disease, pulmonary diseases, and atherosclerotic cardiovascular disease.

Hematologic malignancies profoundly disrupt the host immune system well before any therapeutic intervention is initiated. Diseases such as leukemia, lymphoma, and multiple myeloma induce alterations in both innate and adaptive immunity, leading to compromised neutrophil function, impaired lymphocyte activity, and decreased mucosal barrier integrity, including within the oral cavity. These immunological impairments create an environment conducive to systemic low-grade inflammation and increased susceptibility to infections, which collectively influence the composition and stability of the oral microbiota [7]. Recent research has begun to unravel how these malignancies and their associated immune dysfunctions reshape the oral microbial communities [8,9]. For instance, studies have documented a marked reduction in microbial diversity and a shift towards dominance by opportunistic pathogens in patients with hematologic malignancies, even prior to the initiation of chemotherapy or immunotherapy [10]. Such dysbiotic changes are often characterized by a decline in beneficial commensals such as *Streptococcus* and *Neisseria* species, coupled with an overgrowth of potentially harmful bacteria like *Staphylococcus* and *Pseudomonas* [11]. These alterations are further exacerbated by factors common in hematologic patients, including neutropenia, mucosal barrier damage, and the widespread use of antibiotics or immunosuppressive treatments [12].

Longitudinal studies in stem-cell transplant recipients show persistent oral-microbiome disruption, with incomplete recovery even weeks after treatment [13]. This prolonged dysbiosis has clinical ramifications, as it correlates with increased risks of oral mucositis, opportunistic infections, and heightened systemic inflammatory status [13]. The interplay between microbial imbalance and immune dysregulation is particularly critical in the context of immunotherapy. While immunotherapeutic agents, including immune checkpoint inhibitors and CAR-T cell therapies, aim to enhance anti-tumor immunity, they may inadvertently amplify underlying immune disturbances and exacerbate oral microbial dysbiosis [14]. This dual impact not only predisposes patients to local complications such as mucosal inflammation and infection but may also influence systemic treatment outcomes by modulating the host’s immune [15].

Moreover, growing evidence suggests that the oral cavity and the gut are interconnected through the so-called oral–gut axis, a bidirectional communication pathway linking oral microbial communities with intestinal microbiota and systemic immune responses [16]. The oral–gut axis is a bidirectional communication pathway that links the oral and gut microbiota and influences systemic immune responses.

Dysbiosis in the oral cavity can influence gut microbial composition through swallowing of saliva and translocation of bacteria, while alterations in the gut microbiome can, in turn, affect oral microbial homeostasis via immune-mediated mechanisms and metabolic signaling [17].This crosstalk has significant implications for patients undergoing immunotherapy, as gut microbial composition has been shown to modulate treatment efficacy and the risk of immune-related adverse even [18]. Therefore, oral microbial dysbiosis may not only contribute to local complications but also impact systemic immune responses and the overall effectiveness of cancer immunotherapy through its interaction with the gut microbiome [19].

Based on evidence linking periodontitis-associated oral dysbiosis to systemic inflammatory conditions and considering the bidirectional interactions of the oral-gut axis, this study aims to investigate the impact of immunotherapeutic treatments, including immune checkpoint inhibitors and CAR-T cell therapy, on the oral and intestinal microbial communities of patients with hematological malignancies. Specifically, we intend to evaluate how these therapies modulate immune cell populations in peripheral blood and how these changes may correlate with alterations in the oral-gut microbial composition. Ultimately, this study aims to provide insights into the interplay between immune dysregulation, microbial imbalance, and therapy-related toxicities, highlighting potential targets for host and microbiome modulatory interventions.

## 2. Materials and Methods

### 2.1. Protocol and Registration

This review was conducted in accordance with the PRISMA 2020 guidelines [20]. No formal protocol was registered in PROSPERO or other registries. Any methodological deviations from the initial plan are transparently described within this section.

### 2.2. Eligibility Criteria

Studies were considered eligible if they included original research articles, clinical trials, cohort or case–control studies, or systematic reviews investigating the relationship between immune dysregulation, oral or gut microbiota alterations, and periodontal outcomes in patients with hematologic malignancies undergoing immunotherapy. Eligible populations encompassed both adult and pediatric patients diagnosed with hematologic malignancies—such as leukemia, lymphoma, or multiple myeloma—who were treated with immunotherapeutic or immunomodulatory agents, including monoclonal antibodies, immune checkpoint inhibitors, CAR-T cell therapies, immunomodulatory drugs (IMiDs), interferons, and BTK or JAK inhibitors.

The main outcomes of interest were changes in the oral or gut microbiome, periodontal parameters, and immunological responses associated with host–microbiome interactions.

Studies were excluded if they focused exclusively on solid tumors or conditions unrelated to the oral cavity, or if they did not provide clinical data relevant to periodontal health or microbiome modulation. Narrative reviews, editorials, commentaries, conference abstracts, and in vitro or animal studies were also excluded. Only studies published in English or Italian were considered, unless a reliable English translation was available.

### 2.3. Information Sources and Search Strategy

A comprehensive literature search was carried out in the electronic databases PubMed, Scopus, Web of Science, and Google Scholar. The search strategy was designed to identify studies exploring the interplay between cancer immunotherapy, immune regulation, periodontal inflammation, and microbiome modulation in patients with hematologic malignancies. To ensure methodological rigor and reproducibility, a combination of controlled vocabulary (MeSH terms) and free-text keywords was used.

### 2.4. Study Selection

**Figure 2:**
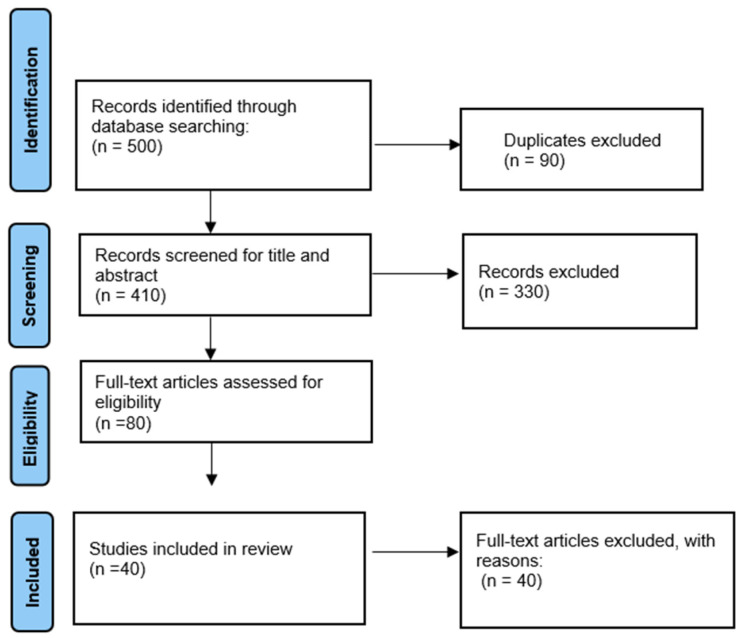
PRISMA flowchart showing the selection process for included studies.

Following the removal of duplicate records, titles and abstracts were screened to assess relevance based on the predefined eligibility criteria. Full-text articles of those deemed potentially eligible were then retrieved and evaluated in detail, with decisions to include or exclude studies documented along with clear reasons for exclusions. The selection process adhered to the PRISMA 2020 workflow and is summarised in the PRISMA flow diagram (Figure 2), which shows the numbers of records identified, screened, excluded, and finally included in the qualitative synthesis.

## 3. Periodontal Implications of Immunotherapy

Immunotherapy has transformed the therapeutic landscape of hematologic malignancies, offering unprecedented improvements in survival and long-term remission [21]. Unlike chemotherapy and radiotherapy, immunotherapies work by restoring or enhancing immune responses against malignant cells [22].

The most widely applied approaches include immune checkpoint inhibitors (ICIs), which block inhibitory pathways such as PD-1/PD-L1 and CTLA-4 to restore effector T-cell activity, and chimeric antigen receptor (CAR)-T cell therapies, in which autologous T lymphocytes are genetically modified to selectively recognize tumor-associated antigens [23]. Additional modalities, including monoclonal antibodies, bispecific T-cell engagers, and cytokine-based therapies, further expand the therapeutic armamentarium [24]. While these treatments have demonstrated remarkable clinical efficacy, they also profoundly reshape immune homeostasis, introducing new complexities in the management of oral and periodontal health.

In particular, ICIs and CAR-T therapies can significantly alter immune surveillance and regulatory pathways, with potential consequences for periodontal tissues that extend beyond those typically seen with traditional cancer treatments [25].

By reducing self-tolerance and promoting T-cell hyperactivation, checkpoint blockade can amplify inflammatory cascades within the gingival microenvironment. Similarly, CAR-T therapies are frequently accompanied by intense cytokine release (e.g., IL-6, TNF-α, IFN-γ), which promotes osteoclastogenesis, connective tissue degradation, and alveolar bone resorption [26]. Moreover, innate immune alterations, particularly involving neutrophils and monocyte/macrophage lineages, may further disturb the balance between protective defense and destructive inflammation [27].

Immunotherapy-related immune changes, together with antibiotics and mucosal toxicity, often promote oral dysbiosis [28]. This, in turn, synergizes immune hyperreactivity to accelerate periodontal breakdown. In this context, even a modest microbial load can lead to significant tissue destruction due to immune-driven mechanisms [29]. Clinically, patients receiving immunotherapy may present with atypical or rapidly progressive periodontal manifestations, including persistent gingival inflammation, accelerated attachment loss, impaired healing after periodontal procedures, and refractory responses to conventional therapy [30].

The activation of immune responses induced by these therapies may therefore amplify local periodontal inflammation, particularly in the presence of pre-existing disease, and oral tissues may become unintended targets of heightened immune activity [31].

These findings highlight the need for early periodontal risk assessment and tailored preventive care [32] Approaches aimed at stabilizing the oral microbiome, reducing inflammatory burden, and integrating host-modulation therapies may help mitigate periodontal morbidity, thereby supporting both oral and systemic health in patients undergoing advanced oncologic treatment [33] (Table 1).

### 3.1. Effects of Immune Checkpoint Inhibitors and CAR-T Cells on Periodontal Tissues

The advent of immunotherapy has revolutionized cancer treatment but also brought new challenges related to immune-related adverse events (irAEs), including those affecting the oral cavity. Immune checkpoint inhibitors (ICIs), such as anti-PD-1 and anti-CTLA-4 antibodies, have been associated with a range of oral complications, including stomatitis, xerostomia, lichenoid reactions, and notably, exacerbation of periodontal disease [34]. A recent retrospective cohort study found that patients treated with ICIs had a significantly higher risk of developing periodontitis compared to non-treated patients, with incidence rates of 55.3 vs. 25.8 per 100 person-years, respectively. Moreover, PD-1 and PD-L1 inhibitors were associated with increased hazard ratios for periodontitis of 2.0 and 2.5, respectively [22].

Beyond T-cell hyperactivation, ICIs also affect innate immune populations, including neutrophils and monocyte/macrophage lineages, thereby amplifying oxidative stress, osteoclastogenesis, and alveolar bone resorption [35].

These oral side effects are thought to stem from immune dysregulation caused by ICIs, which disrupt the delicate balance between the host immune response and the oral microbiome. For instance, increased expression of PD-1 and PD-L1 proteins has been observed in periodontal tissues from patients with periodontitis, suggesting that pathogens such as *Porphyromonas gingivalis* may exploit these immune checkpoints to evade immune surveillance [36].

Immunotherapy may induce oral dysbiosis, especially with concurrent antibiotic use, promoting pro-inflammatory species and reducing diversity [37].

CAR-T cell therapy, while highly effective for hematologic malignancies, also triggers profound immune activation, leading to systemic cytokine release syndrome (CRS) and immune effector cell-associated neurotoxicity syndrome (ICANS). Although oral complications have been less extensively studied in this context, emerging case reports highlight occurrences of oral mucosal lesions and inflammation following CAR-T therapy, indicating the need for careful periodontal monitoring [38].

These findings are biologically plausible, since the cytokine storm associated with CRS—particularly elevated IL-6, IL-1β, and TNF-α—may enhance extracellular matrix degradation and delay periodontal wound healing [39].

From a clinical perspective, early periodontal assessment, preventive strategies targeting dysbiosis, and host-modulation therapies should be considered as adjunctive measures within oncologic care pathways [40].

Overall, both ICIs and CAR-T therapies have important implications for periodontal health through complex immune-mediated mechanisms. Awareness of these effects and proactive oral health management integrated within oncologic care pathways may help mitigate periodontal complications, improve patient quality of life, and optimize systemic treatment outcomes.

### 3.2. Clinical Manifestations and Periodontal Considerations in Immunotherapy-Treated Patients

Although most immunotherapy-related oral manifestations are adverse, certain immune-modulating effects may also exert indirect benefits. By enhancing immune surveillance and reducing microbial immune evasion, checkpoint inhibition might transiently suppress specific pathogenic species or modulate dysbiotic communities toward a more balanced composition. However, these potential benefits are usually outweighed by excessive inflammatory activation, which can trigger or exacerbate mucosal and periodontal damage [38].

Typical oral disorders during immunotherapy include mucositis, xerostomia, lichenoid or ulcerative lesions, gingival bleeding, and candidiasis.

Recent systematic reviews and meta-analyses have reported that immune checkpoint inhibitors (ICIs) are associated with a high prevalence of oral immune-related adverse events, with xerostomia and mucositis being the most common [30].

Periodontitis has also been recognized as a potential immune-related adverse event in ICI-treated patients, with hazard ratios ranging from 2.0 to 2.5 compared to non-treated individuals [22].

These conditions are often manageable through basic supportive measures, like meticulous oral hygiene, professional cleaning, topical anti-inflammatory or antifungal agents, saliva substitutes, and nutritional counseling which can markedly improve comfort and reduce secondary complications [41]. In particular, maintaining microbial homeostasis and controlling local inflammation may mitigate both oral and systemic sequelae of therapy.

Importantly, the baseline periodontal condition significantly influences both the susceptibility and severity of oral side effects. Pre-existing periodontal inflammation amplifies local cytokine release, delays mucosal healing, and may act as a bacterial reservoir during therapy-induced immunosuppression, increasing the risk of systemic infection or bleeding [41]. Conversely, maintaining periodontal health through preventive and supportive care before and during immunotherapy may help stabilize local inflammation, enhance mucosal resilience, and improve overall treatment tolerance [42]. Therefore, comprehensive periodontal evaluation and individualized preventive strategies should be integral components of supportive care in patients receiving immunomodulatory therapies for hematological malignancies.

Accordingly, periodontal therapy in hematologic patients can play a preventive role before initiating immunomodulatory treatment, helping to reduce the risk of oral and systemic complications, and a supportive role during therapy, mitigating local adverse effects, stabilizing inflammation, and improving treatment tolerance and patient quality of life.

### 3.3. Periodontitis as a Source of Systemic Inflammation in Immunocompromised Patients

In immunocompromised individuals, particularly those affected by hematologic malignancies, periodontitis is not confined to the oral cavity but acts as a chronic source of systemic immune activation. In these patients, the impaired ability to regulate inflammatory responses allows for the dissemination of pro-inflammatory mediators such as interleukin-1β, tumor necrosis factor-alpha, and IL-6 into the bloodstream, amplifying systemic immune stress [43]. Unlike in immunocompetent hosts, where local containment mechanisms are more effective, the sustained inflammatory burden from periodontal tissues may interfere with critical immunologic and hematopoietic pathways.

Recent study indicates that microbial products and cytokines originating from periodontal lesions can reach the bone marrow and disrupt the architecture and function of the hematopoietic niche. This may alter the differentiation and mobilization of stem and progenitor cells, contributing to immune imbalance and increasing vulnerability to infections and treatment-related complications in patients undergoing chemotherapy or immunotherapy [44].

Chronic peripheral inflammation, as seen in periodontitis, has also been linked to skewed myelopoiesis and reduced lymphoid output, both of which can impair the body’s ability to mount effective anti-tumor responses [45].

The influence of periodontal inflammation may extend to the oral–gut–systemic axis, an emerging concept in cancer immunology. Inflammatory signals and bacterial metabolites originating in the oral cavity can affect gut barrier integrity and modulate intestinal microbiota composition, promoting systemic immune activation and low-grade endotoxemia [18]. This is particularly concerning in immunocompromised patients, in whom mucosal barrier dysfunction and microbiome instability are already prevalent due to disease or treatment.

Periodontal evaluation and management into the supportive care of patients with hematologic malignancies may reduce systemic immune activation, enhance mucosal immunity, and potentially support better therapeutic outcomes.

## 4. The Oral–Gut Axis During Periodontitis and Undergoing Hematologic Immunotherapy

The oral–gut axis is a bidirectional pathway linking the oral cavity and gut and influencing local and systemic immune responses. Dysbiosis in the oral microbiota, often resulting from periodontal diseases, can lead to microbial translocation to the gut, thereby affecting gut microbiota composition and potentially contributing to systemic inflammation and immune modulation [16]. At the oral level, dysbiosis is characterized by an increase in proteolytic, anaerobic, and asaccharolytic species such as *Porphyromonas gingivalis* (*P. gingivalis*), *Tannerella forsythia*, and *Treponema denticola*, accompanied by a reduction in commensal genera such as *Streptococcus* and *Actinomyces* [46].

This microbial shift enhances the local production of pro-inflammatory cytokines (IL-1β, IL-6, TNF-α) and matrix metalloproteinases, promoting connective-tissue destruction, alveolar bone resorption, and the loss of periodontal homeostasis [47].

When oral pathogens or their metabolites translocate to the gastrointestinal tract, they induce significant ecological and immunological alterations. Gut microbial diversity is often reduced, with enrichment of pro-inflammatory taxa such as Enterobacteriaceae and depletion of short-chain fatty acid (SCFA)-producing bacteria, including *Faecalibacterium prausnitzii* and *Roseburia* spp. [48]. This imbalance compromises mucosal-barrier integrity, increases intestinal permeability (“leaky gut”), and facilitates endotoxin translocation into the bloodstream, resulting in systemic inflammation and altered bile-acid metabolism involving bile-salt hydrolase (BSH) activity and farnesoid X receptor (FXR) signaling, mechanisms that perpetuate metabolic dysregulation and chronic immune activation [47].

This interplay is of particular concern in patients with hematologic malignancies undergoing immunotherapy, as both the disease and treatment modalities can exacerbate microbial imbalances and immune dysregulation [49].

Periodontitis is characterized by pathogens like *P. gingivalis* and *Fusobacterium nucleatum* (*F. nucleatum*) playing pivotal roles. These microorganisms can translocate to the gastrointestinal tract via the oral–gut axis, influencing gut microbiota composition and potentially contributing to systemic inflammation and immune modulation [50]. *P. gingivalis* is able to evade host immune surveillance and promote periodontal tissue destruction through the release of virulence factors such as lipopolysaccharides and proteases [51].

In addition, this pathogen can manipulate both innate and adaptive immune responses. Specifically, *P. gingivalis* expresses cysteine proteases known as gingipains, which degrade cytokines and complement components, thereby dampening host antibacterial defenses. It also produces atypical lipopolysaccharide (LPS) structures that differentially activate Toll-like receptors (TLR2/TLR4), modulating the inflammatory response toward a chronic, low-grade profile. Furthermore, through its fimbriae and outer membrane vesicles, *P. gingivalis* interferes with dendritic-cell maturation and promotes the polarization of T cells toward a Th17/Treg imbalance, contributing to immune evasion and persistent infection [52]. Once translocated to the intestine, these pathogens can disrupt mucosal barrier integrity and alter microbial ecology. In particular, *F. nucleatum* has been shown to damage the intestinal epithelium and enhance local inflammatory responses [53]. Moreover, dysbiosis of the gut microbiota may further affect bile-salt metabolism and FXR signaling, linking periodontal pathogens to intestinal chronic inflammation and systemic immune dysregulation [54] (Figure 2)

In the context of hematologic malignancies, such as leukemia and lymphoma, the immune system is often compromised, leading to increased susceptibility to infections and further perturbations in microbial communities. Immunotherapies, including immune checkpoint inhibitors and CAR-T cell therapies, aim to enhance anti-tumor immunity but may inadvertently amplify underlying immune disturbances and exacerbate oral microbial dysbiosis [16].

The implications of these interactions are profound. Oral microbial dysbiosis and subsequent gut-microbiota alterations can modulate both the efficacy and toxicity of immunotherapy. Certain gut microbiota compositions have been associated with improved responses to immunotherapy, while others may contribute to adverse effects. Therefore, understanding the dynamics of the oral–gut microbiome axis in this patient population is crucial for optimizing therapeutic outcomes and minimizing complication [55].

The oral-gut axis plays a significant role in the health and disease outcomes of individuals with periodontitis and hematological malignancies undergoing immunotherapy. Alterations in this axis may drive systemic inflammation, modulate immune responses, and impact treatment efficacy [56]. Future research focused on modulating this axis via host-modulation therapies, microbiome-targeted interventions, or combined periodontal oncologic strategies may offer new opportunities to improve clinical outcomes in this vulnerable population [57].

## 5. Host-Modulation Therapies in Periodontology

Periodontal disease is increasingly recognized not merely as a bacterial infection but as a chronic inflammatory condition driven by a dysregulated host response to microbial dysbiosis. This shift in understanding has led to the development of host-modulation therapies (HMTs), a class of adjunctive treatments that aim to regulate the destructive aspects of the immune-inflammatory cascade without compromising essential immune defense mechanisms [58].

Recent studies have highlighted that host-modulation therapies can also influence innate immune cell functions, including neutrophils, monocytes, and macrophages, thereby reducing oxidative stress, modulating cytokine profiles, and limiting tissue-destructive inflammation [59].

These therapies target key elements of the pathogenesis of periodontitis, including pro-inflammatory cytokines, matrix-degrading enzymes, oxidative stress, and the resolution of inflammation.

Emerging evidence also suggests that certain HMTs can interact with the oral microbiome, promoting microbial balance while simultaneously dampening host-mediated tissue damage, thus reinforcing periodontal homeostasis [60].

The goal is to reduce connective tissue breakdown, inhibit alveolar bone resorption, and support the re-establishment of periodontal homeostasis. Unlike conventional approaches focused on bacterial elimination alone, HMTs recognize the critical interplay between the host immune system and the resident microbiota, offering a more biologically integrative perspective [41].

This integrated approach is particularly relevant in patients undergoing systemic treatments, including immunotherapy, where host response modulation may help mitigate exacerbations of periodontal inflammation and improve overall oral-systemic outcomes [61].

### 5.1. Mechanisms and Clinical Rationale

In periodontitis, it is not only the bacteria that cause damage to the gums and surrounding tissues, but also the body’s own immune system. This leads to chronic inflammation, persistent neutrophil infiltration, and elevated production of pro-inflammatory cytokines such as IL-1β, TNF-α, and IL-17. The continued activation of osteoclastogenic pathways, particularly through the RANKL/OPG imbalance, results in progressive alveolar bone loss [62].

**Figure 3:**
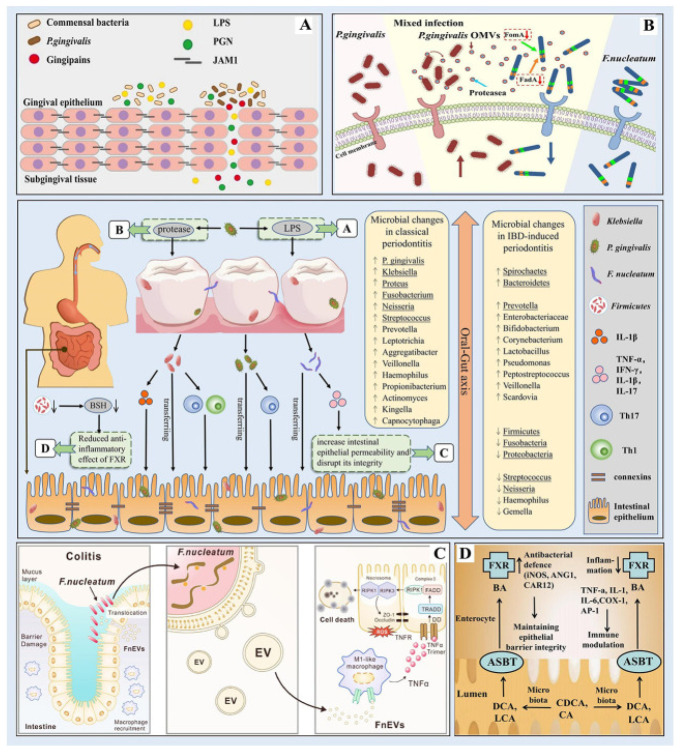
Microbial correlation between periodontitis and inflammatory bowel diseases (IBD). In periodontitis, the oral flora is altered and *P. gingivalis* evades host immune defense to destroy periodontal tissue by releasing virulence factors such as proteases and lipopolysaccharides; alterations in the composition of the intestinal microbiota leading to changes in bacterial metabolites such as BSH may play an important role in the pathogenesis of IBD; through the oral-gut axis, periodontal pathogenic bacteria such as *Klebsiella*, *P. gingivalis*, and *F. nucleatum* can ectopically colonize the intestine and disrupt the intestinal barrier thus leading to intestinal ecological dysregulation and chronic inflammation. (**A**) *P. gingivalis* produces virulence factors such as LPS; (**B**) *P. gingivalis* produces virulence factors such as proteases; (**C**) *F. nucleatum* destroys the intestinal mucosa; (**D**) The role of FXR in IBD. Reproduced from [50] under Creative Commons CC-BY licence agreement.

When the immune response becomes exaggerated or poorly regulated, it leads to chronic inflammation that destroys bone and connective tissue. HMT are designed to help the body control this destructive inflammation, without suppressing the immune system entirely [58] (Figure 3).

Scientific research has shown that inflammation and microbial imbalance (dysbiosis) feed into each other. As inflammation progresses, tissue damage releases nutrients that favor the growth of certain bacteria, which in turn worsen the inflammation. Some bacteria—like *Porphyromonas gingivalis*—can actively manipulate the immune response, helping create a disease-promoting environment even when present in low numbers [63].

Recent advances in periodontal immunology have identified several host-derived molecules that regulate inflammation and promote resolution. Among these, Developmental Endothelial Locus-1 (Del-1) plays a key role in controlling neutrophil recruitment. Del-1 interferes with the binding of LFA-1 on leukocytes to ICAM-1 on endothelial cells, thus limiting neutrophil extravasation into the periodontal tissue [64]. Moreover, Del-1 is not only anti-inflammatory but also pro-resolving. It promotes the clearance of apoptotic cells by macrophages (a process known as efferocytosis), facilitating the return to tissue homeostasis. Importantly, therapeutic administration of Del-1 in human with induced periodontitis significantly reduced inflammation and inhibited osteoclastogenesis and bone loss, providing strong translational evidence for its clinical relevance [65].

While, in animal models, Del-1 deficiency has been shown to result in enhanced neutrophilic inflammation, elevated IL-17 expression, and more severe periodontal bone loss [62].

**Figure 4:**
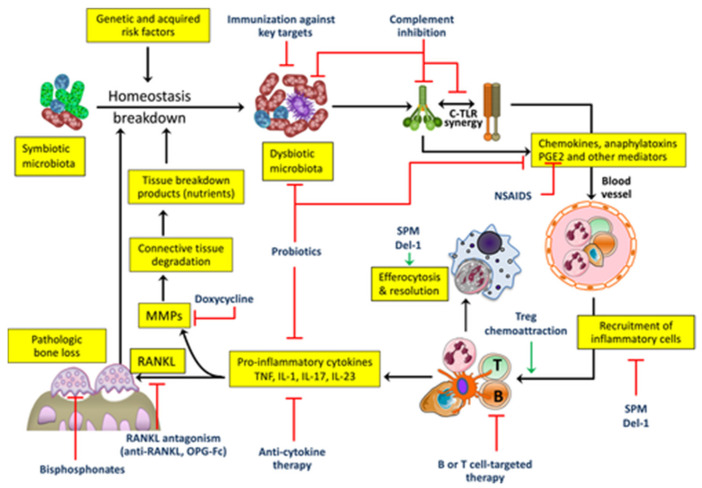
Targets for host-modulation interventions in periodontitis. Periodontitis arises from the disruption of host-microbe homeostasis in susceptible individuals, leading to dysbiosis and destructive inflammation that not only activates osteoclastogenesis and bone loss but also provides nutrients (tissue breakdown products) that enable the dysbiotic microbiota to grow and persist. Shown are important therapeutic targets and potential interventions, most of which are currently at an experimental stage (see the text for details). C, complement; Del-1, development endothelial locus-1; IL, interleukin; MMPs, matrix metalloproteinases; NSAIDs, nonsteroidal anti-inflammatory drugs; OPG-Fc, osteoprotegerin (tumor necrosis factor receptor superfamily member 11B) fused to the Fc part of IgG; PGE2, prostaglandin E2; RANKL, receptor activator of nuclear factor-kappaB ligand (tumor necrosis factor ligand superfamily member 11); SPM, specialized pro-resolving mediators; TLR, toll-like receptor; TNF, tumor necrosis factor; Treg, regulatory T-cell. From Hajishengallis et al. 2020 [58], with permission.

In clinical contexts where immune function is already compromised—such as patients undergoing immunotherapy for hematologic malignancies—host-modulation may be particularly beneficial. By reducing the local inflammatory burden in the periodontium, these therapies could help limit systemic immune activation, reduce collateral tissue damage, and potentially improve treatment tolerance or outcomes (Figure 4).

### 5.2. Sub-Antimicrobial Dose Doxycycline (SDD) and MMP Inhibition

Sub-antimicrobial dose doxycycline (SDD) refers to a formulation of doxycycline administered at doses (typically 20 mg twice daily) that are significantly lower than those required to exert antibacterial effects. Approved by the FDA for use in chronic periodontitis, SDD exerts its therapeutic benefits not by targeting microbial pathogens directly, but by modulating the host immune response, particularly through the inhibition of matrix metalloproteinases (MMPs) involved in connective tissue breakdown [66].

MMPs, especially MMP-8, MMP-9 and MMP-13, are collagen-degrading enzymes released by neutrophils and other inflammatory cells during the immune response to periodontal biofilms. In chronic periodontitis, persistent overexpression of these enzymes contributes to the degradation of the extracellular matrix, loss of periodontal attachment, and alveolar bone resorption. SDD has been shown to downregulate MMP expression and activity without affecting the normal microbial flora or inducing antimicrobial resistance [67] (Table 2).

Beyond its local periodontal effects, SDD may also reduce systemic inflammatory mediators such as C-reactive protein (CRP), interleukin-6 (IL-6), and tumor necrosis factor-alpha (TNF-α), particularly in systemically vulnerable populations like individuals with diabetes or cardiovascular disease. These findings suggest a potential role for SDD in managing chronic inflammatory burden not only locally in periodontal tissues, but also at the systemic level.

Given its proven safety and ability to reduce inflammation and tissue breakdown, sub-antimicrobial dose doxycycline (SDD) is a useful addition in treating periodontal disease, especially in immunocompromised patients or those receiving immunotherapy. SDD helps protect the tissues by blocking enzymes that cause damage, without disturbing the natural balance of oral bacteria. This makes it a valuable option for managing periodontal health in medically complex patients, supporting both local tissue preservation and overall immune balance [68].

### 5.3. Omega-3 Fatty Acids and Resolution of Inflammation

**Figure 5:**
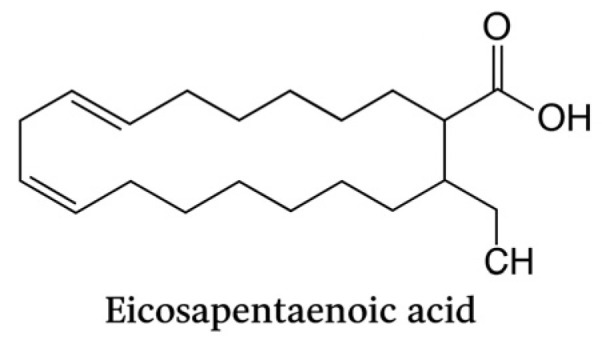
Chemical structure of eicosapentaenoic acid (EPA). Generated with artificial intelligence (OpenAI DALL·E, 2025).

**Figure 6:**
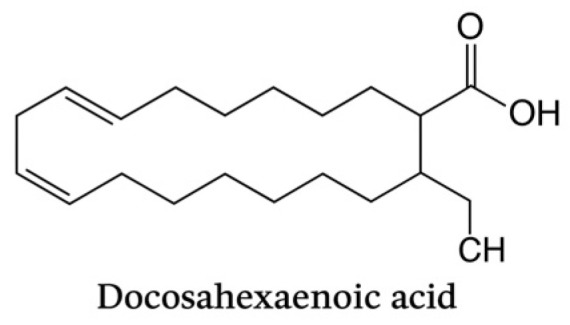
Chemical structure of docosahexaenoic acid (DHA). Generated with artificial intelligence (OpenAI DALL·E, 2025).

Omega-3 polyunsaturated fatty acids (n-3 PUFAs), particularly eicosapentaenoic acid (EPA) and docosahexaenoic acid (DHA), play a crucial role in promoting the resolution of inflammation, a process now recognized as active and highly regulated, rather than passive. According to Serhan et al., EPA and DHA are precursors to a family of specialized pro-resolving mediators (SPMs), including resolvins (RvE and RvD series), protectins, and maresins, which are endogenously biosynthesized during the resolution phase of inflammation [24] (Figure 5 and Figure 6).

**Figure 7:**
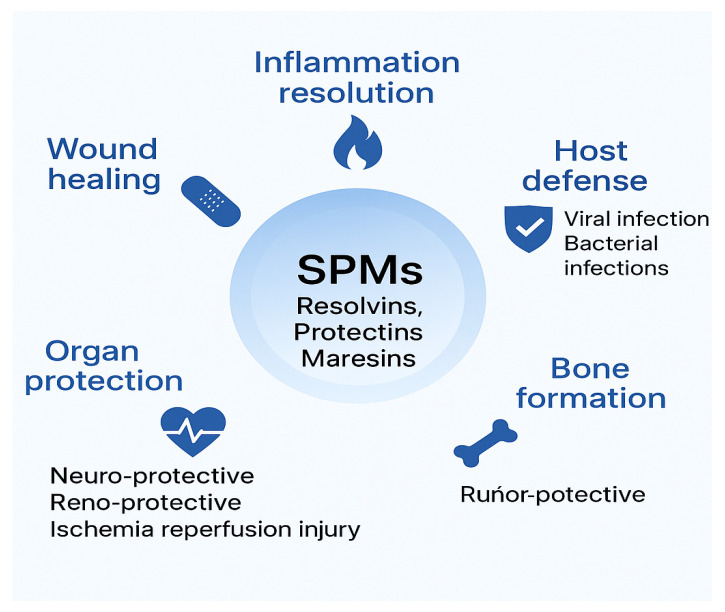
Biological functions of specialized pro-resolving mediators (SPMs), including resolvins, protectins, and maresins. These lipid mediators actively contribute to the resolution of inflammation, modulation of pain, wound healing, tissue regeneration, bone formation, and adipose tissue regulation. SPMs enhance host defense against infections and exert organ-protective effects, including neuroprotection, renoprotection, and cardioprotection, thereby highlighting their therapeutic potential in the context of cancer and systemic diseases. Generated with artificial intelligence (OpenAI DALL·E, 2025).

SPMs do not block inflammation; they actively clear neutrophils, enhance macrophage phagocytosis, and restore tissue homeostasis without impairing host defense. This distinction is particularly relevant in chronic diseases like periodontitis, where inflammation is prolonged and tissue destructive (Figure 7).

In periodontal therapy, adjunctive use of omega-3 fatty acids has been associated with clinical improvements, including reduced probing depths and bleeding on probing, along with significant reductions in inflammatory markers such as IL-1β and TNF-α [69].

The biological rationale behind these outcomes is closely tied to the pro-resolving actions of SPMs, which modulate the immune response without triggering immunosuppression. Importantly, in medically compromised individuals—such as those undergoing immunotherapy for hematological malignancies, this resolution-promoting action may help control excessive immune activation locally in the periodontium while preserving systemic immune competence [70].

In patients with hematological malignancies, omega-3 fatty acids may exert additional beneficial effects on oral and periodontal health. These patients frequently experience oral mucosal inflammation, dysbiosis, delayed wound healing, and exaggerated immune responses due to chemotherapy, immunotherapy, or bone marrow suppression. Omega-3 supplementation can attenuate these complications by reducing oxidative stress and neutrophil hyperactivity, promoting epithelial repair, and restoring microbial balance within the oral cavity [69].

Moreover, SPMs derived from EPA and DHA have been shown to enhance mucosal barrier integrity and limit systemic cytokine overproduction, which may decrease the incidence or severity of oral mucositis and secondary infections [71]. Therefore, in hematological patients undergoing immunotherapy, omega-3 fatty acids might contribute to maintaining periodontal stability, mitigating oral inflammation, and supporting mucosal resilience, without compromising anti-tumor immune activity [72].

Eicosapentaenoic acid (C20:5, n-3) is a long-chain omega-3 polyunsaturated fatty acid with five cis double bonds. It represents a key precursor of specialized pro-resolving mediators (SPMs), including resolvins and protectins, which exert anti-inflammatory and pro-resolutive effects in periodontal tissues.

Docosahexaenoic acid (C22:6, n-3) is a long-chain omega-3 polyunsaturated fatty acid characterized by six cis double bonds. It contributes to membrane fluidity and serves as a precursor of SPMs with potent immunomodulatory activity, playing a pivotal role in the resolution of periodontal inflammation.

## 6. Oral Microbiome-Targeted Strategies

In patients with hematologic malignancies, periodontal interventions must account for immunosuppression, cytopenias, and increased susceptibility to infections and bleeding. Both conventional mechanical therapy and emerging microbiome-targeted strategies should be adapted to minimize risks while supporting oral and systemic health [73].

The oral microbiome contributes to periodontal disease by triggering local inflammation and influencing systemic immune responses which involves some endothelial ecographic mediators [74]. Recent mechanistic studies have demonstrated that microbial dysbiosis in the oral cavity can influence systemic immunity by modulating the production of pro-inflammatory cytokines and altering the balance between regulatory and effector T cells. These immune perturbations can exacerbate both local periodontal destruction and distant inflammatory responses, including those affecting the cardiovascular and hematopoietic systems. In particular, in patients with hematologic malignancies, even subtle shifts in oral microbial composition may have amplified consequences due to the underlying immune dysregulation and concurrent therapies that compromise host defense mechanisms [75].

In patients undergoing immunotherapy for hematological malignancies, the oral microbial environment may become particularly unstable due to immune suppression, mucosal damage, or altered host-microbiota interactions.

This instability creates a window of opportunity for opportunistic pathogens to proliferate, which can trigger exaggerated inflammatory responses and further compromise mucosal integrity. Longitudinal studies have shown that immune checkpoint inhibitors and CAR-T therapies can indirectly affect microbial composition, favoring pathobionts that exploit immune dysregulation while simultaneously reducing populations of commensal, health-associated microbes. Consequently, patients may experience accelerated periodontal tissue breakdown, delayed wound healing, and higher susceptibility to oral mucositis, emphasizing the need for microbiome-targeted prophylactic interventions [76].

The ecological and host-modulating approach represents a paradigm shift in periodontal therapy. It focuses on restoring microbial balance and regulating the host immune-inflammatory response rather than merely eliminating pathogenic bacteria. This strategy acknowledges that tissue destruction results not only from microbial overgrowth but also from dysregulated host immunity. By stabilizing microbial communities, controlling local inflammation, and enhancing tissue resilience, such interventions aim to prevent or mitigate periodontal damage while supporting systemic health, particularly in immunocompromised patients [77].

Recent studies have shifted from purely antimicrobial approaches toward microbiome-modulating therapies, aiming not only to reduce pathogenic species but also to restore microbial balance and promote symbiosis.

These microbiome-targeted strategies encompass probiotics, prebiotics, postbiotics, and synbiotics, all designed to selectively enhance the growth and metabolic activity of beneficial bacteria. Beyond direct antimicrobial effects, these interventions can influence local immune signaling pathways, enhance epithelial barrier function, and promote the production of anti-inflammatory metabolites such as short-chain fatty acids. In hematologic patients, these microbiome-modulating interventions may be particularly valuable in reducing oral inflammation, preventing opportunistic infections, and supporting mucosal barrier integrity during immunosuppressive therapies [78].

Clinical evidence indicates that integrating these therapies with standard periodontal care can reduce clinical attachment loss, decrease probing depths, and attenuate inflammatory biomarkers in saliva and gingival crevicular fluid, particularly in high-risk or immunocompromised populations [79].

While conventional periodontal treatments like mechanical debridement, surgical interventions, and local antiseptics, effectively reduce bacterial load and control local inflammation, they do not directly modulate the host immune response or restore microbial balance. In contrast, microbiome-targeted strategies aim to re-establish a resilient oral microbial ecosystem and regulate immune-inflammatory pathways, providing complementary benefits that may enhance long-term periodontal and systemic outcomes. Limitations include variability in clinical evidence, strain specificity, and the need for individualized protocols.

This shift is rooted in evidence that a resilient, eubiotic oral microbiota is crucial for maintaining immune tolerance and mucosal homeostasis, especially in immunocompromised individuals [80]. Eubiosis refers to a balanced, health-associated microbial community that supports immune and mucosal homeostasis.

New strategies include probiotics, prebiotics, and postbiotics, designed to support beneficial microbial communities and suppress overgrowth of pathogenic taxa. These approaches may enhance periodontal healing and reduce systemic inflammatory burden without the drawbacks of conventional antimicrobial overuse [81].

Importantly, these therapies represent a paradigm shift toward a more holistic management of periodontal disease, emphasizing the restoration of a resilient microbial ecosystem rather than indiscriminate microbial eradication. For patients undergoing chemotherapy or immunotherapy, local antimicrobial delivery can reduce systemic drug exposure and lower infection risk while maintaining control of subgingival biofilms [73].

In patients receiving immunotherapy, early integration of microbiome-modulating interventions may not only protect oral tissues but also modulate systemic immune responses, potentially enhancing treatment tolerability and clinical outcomes. Overall, in hematologic patients, an integrated approach combining adapted mechanical therapy, microbiome-modulating strategies, and host-modulating interventions is crucial to maintain periodontal health, reduce systemic inflammatory burden, and enhance tolerability of oncologic treatments [73].

Future research is focusing on personalized microbiome-based strategies, including strain-specific probiotics and tailored prebiotic formulations, to optimize host-microbe interactions and sustain long-term periodontal and systemic health [82].

### 6.1. Oral Dysbiosis and Its Role in Periodontal Pathogenesis

Periodontitis is a chronic, multifactorial inflammatory disease driven by a dysregulated interaction between the host immune system and the subgingival microbiome [63].

It is no longer understood as the result of infection by a few classical pathogens, but rather as a disease of microbial imbalance shift from a symbiotic, health-associated microbiota to a dysbiotic, inflammation-promoting microbial community [46].

Oral dysbiosis involves the expansion of opportunistic pathogens and reduced microbial diversity, creating chronic inflammation that drives tissue destruction [83].

In periodontal health, the oral microbiota is characterized by high diversity and a predominance of commensal and mutualistic species, which help maintain mucosal immunity and suppress the overgrowth of pathogens. However, in periodontitis, environmental changes—such as inflammation, increased gingival crevicular fluid, and immune alterations—select for opportunistic and proteolytic bacteria, including *Porphyromonas gingivalis*, *Tannerella forsythia*, and *Treponema denticola.* These organisms are capable of subverting the immune response, impairing neutrophil function, and altering the epithelial barrier, creating a self-sustaining inflammatory loop [84].

In oncologic or immunocompromised patients, including those undergoing immune checkpoint inhibitors (ICI) or CAR-T cell therapy, the risk of oral dysbiosis is amplified. These therapies, while enhancing antitumor immunity, can disrupt mucosal homeostasis, reduce salivary flow, and alter neutrophil function, factors that contribute to shifts in microbial composition and increased susceptibility to opportunistic infections and periodontal deterioration. Furthermore, chemotherapy or immunotherapy-induced neutropenia may reduce microbial clearance, facilitating overgrowth of dysbiotic biofilms [85].

Clinically, these patients often present with atypical periodontal progression, increased bleeding tendency, and delayed healing. Additionally, oral dysbiosis may represent a source of systemic immune activation, which is particularly concerning in patients with cancer, as chronic low-grade inflammation can interfere with treatment response, increase the risk of immune-related adverse events (irAEs), and compromise mucosal barrier integrity [86].

Therefore, in immunocompromised patients, monitoring and managing oral dysbiosis becomes essential, not only for preserving periodontal health, but also for supporting systemic immune regulation and minimizing complications associated with anticancer therapies [87].

### 6.2. Probiotics, Prebiotics, and Microbial Balance Restoration

A healthy oral microbiome is essential for preventing and managing periodontal disease, especially in individuals with weakened immune systems. Traditional treatments often focus on eliminating harmful bacteria, but recent advances highlight the importance of supporting beneficial microbes to restore and maintain a balanced oral environment [74]. Recent studies emphasize that interventions targeting the oral microbiome, including probiotics and prebiotics, can modulate local immune responses, reduce oxidative stress, and limit periodontal tissue breakdown, offering a complementary strategy to conventional mechanical therapy [88].

Probiotics and prebiotics have gained attention as innovative, biologically based approaches to modulate the oral microbiota and promote periodontal health.

Probiotics are live microorganisms that, when administered in adequate amounts, confer a health benefit to the host by modulating the microbiota and the immune response. In the oral cavity, probiotic strains such as Lactobacillus reuteri, Streptococcus salivarius, and Bifidobacterium species have shown the ability to inhibit the growth of periodontal pathogens, reduce inflammation, and improve clinical parameters like probing depth and bleeding on probing. These beneficial bacteria compete with pathogens for adhesion sites, produce antimicrobial substances, and stimulate anti-inflammatory cytokines, thereby helping to re-establish a balanced oral ecosystem [89].

Emerging clinical trials suggest that probiotic administration may also enhance mucosal barrier function and reduce systemic inflammatory markers in patients with compromised immunity, including those receiving immunotherapy [90].

While probiotics have demonstrated benefits in modulating the oral microbiota and reducing periodontal inflammation, their use in severely immunocompromised patients requires caution [73]. In patients with profound neutropenia, hematopoietic stem cell transplantation, or active chemotherapy-induced immunosuppression, there is a risk albeit low of bacteremia or fungemia from live microbial supplementation. Current recommendations suggest that probiotics may be considered only in patients with stable immune function, in consultation with the treating hematologist, and using well-characterized strains with documented safety in immunocompromised populations [91]. Administration should ideally be delayed until neutrophil counts have recovered, mucosal integrity is maintained, and systemic infection risk is minimized. In high-risk cases, alternatives such as postbiotics or prebiotics may offer microbial modulation without introducing live organisms [92].

Integrating probiotics under these precautions may help support oral microbial balance and enhance mucosal immunity, while minimizing potential risks in hematologic patients.

Prebiotics are non-digestible compounds that selectively stimulate the growth and activity of beneficial microbes. By providing a favorable nutrient environment, prebiotics can enhance the colonization and function of health-associated bacteria, supporting a resilient microbial community that resists dysbiosis [93].

Prebiotics may synergize with probiotics (synbiotics) to reinforce oral microbial balance, improve clinical periodontal outcomes, and decrease colonization by pathogenic species in high-risk patient populations [94].

For immunocompromised patients, including those undergoing cancer immunotherapy, the use of probiotics and prebiotics may offer additional benefits by strengthening mucosal barriers and reducing systemic inflammatory signals originating from oral dysbiosis [83]. Integrating microbial-targeted strategies with standard periodontal care may therefore provide a holistic approach, particularly in oncology patients, by supporting both oral and systemic health [95].

### 6.3. Local Antimicrobial Protocols

Local antimicrobials are used alongside mechanical therapy to suppress pathogens in periodontal pockets and help restore microbial balance. In addition, important confounding factors are inconsistently reported across studies and may influence both periodontal findings and microbiome alterations.

These interventions include antiseptic mouth rinses, locally delivered antibiotics, and antimicrobial gels or chips, and are especially relevant in cases where systemic treatments are contraindicated or when targeting specific high-risk sites.

The control of subgingival biofilms remains a cornerstone of periodontal therapy, and in recent years, locally delivered antimicrobial protocols have been increasingly explored as adjunctive strategies to support tissue healing and microbial rebalancing. Rather than relying solely on systemic agents, these local interventions aim to deliver therapeutic compounds directly into periodontal pockets, achieving high local concentrations with minimal systemic exposure [96].

Among the most commonly used agents are chlorhexidine chips, doxycycline and minocycline gels, and more recently, biocompatible carriers loaded with natural antimicrobials or anti-inflammatory compounds. Recent study confirmed their utility in reducing pocket depth, improving clinical attachment, and controlling inflammation—particularly when conventional mechanical debridement is insufficient or contraindicated [97].

In patients with altered immune responses—such as those undergoing immunotherapy for hematological malignancies—local antimicrobial strategies may offer additional advantages. By minimizing systemic drug load, they reduce the risk of interactions with cancer treatments and support a more localized modulation of the oral microbiota, which is often dysregulated in these individuals [62].

Furthermore, some delivery systems now incorporate host-modulating compounds, aiming not just to suppress bacteria but to influence the inflammatory environment directly [98].

## 7. Limitations and Future Perspectives

This review has some limitations that should be acknowledged. Most evidence linking immunotherapy, periodontal inflammation, and the oral microbiome in hematologic malignancies comes from small, heterogeneous cohorts, limiting causal inference. Variability in oral health assessments, patient selection, and disease characteristics further reduces data comparability. In addition, few studies have integrated deep immune profiling, which would better elucidate host–microbiome interactions under immunotherapeutic pressure. Future research should include standardized, longitudinal clinical trials combining periodontal, microbiological, and immunological evaluations. Integrative multi-omics analyses (metagenomics, metabolomics, and salivary biomarkers) could clarify the bidirectional relationship between immune modulation and oral dysbiosis. The impact of other immunomodulatory agents such as monoclonal antibodies, IMiDs, BTK and JAK inhibitors, and interferons also warrants investigation. Finally, exploring host-modulation and microbiome-based therapies, including probiotics and postbiotics, through multidisciplinary collaboration between oncologists and periodontists, may guide the development of personalized, evidence-based clinical protocols.

## 8. Conclusions

Periodontal health is a critical determinant of outcomes in patients undergoing immunotherapy for hematologic malignancies. Oral dysbiosis, together with a dysregulated immune response, can worsen local inflammation and influence systemic immune activation, affecting immunotherapy outcomes.

Preventive and therapeutic strategies that stabilize the oral microbiome and modulate host inflammation may therefore support both oral and systemic health. Integrating host-modulation therapies and microbiome-targeted approaches into periodontal care could help reduce inflammatory burden and improve treatment tolerance. Early periodontal assessment, preventive measures, and close monitoring during immunotherapy remain essential. Future research should prioritize personalized microbiome- and host-modulation strategies to enhance clinical outcomes in this vulnerable population.

## Figures and Tables

**Table 1:** Periodontitis immunotherapy strategies. Schematic representation of emerging therapeutic approaches for periodontitis with a focus on host-modulation and innovative adjunctive strategies. Drug-based approaches aim to regulate immune cell subsets and cytokine activity; microbial therapies include probiotic and antibacterial interventions for oral microbiome modulation; stem cell therapies involve periodontal ligament stem cells (PDLSCs), gingival mesenchymal stem cells (GMSCs), and other mesenchymal stem cells (MSCs); gene therapy includes photodynamic therapy (PDT)-associated approaches; other innovative therapies comprise photothermal treatments (PTT), photodynamic protocols (MB-PDT), single-photon modalities, and low-intensity pulsed ultrasound (LIPUS). Modified from Yang et al. 2021 [33], under the terms and conditions of the Creative Commons Attribution (CC BY) license.

Main Category	Therapeutic Approaches
**Drug Therapy**	• Targeting Neutrophils • Targeting Monocytes • Targeting Macrophages • Targeting T Lymphocytes • Targeting Cytokines
**Microbial Therapy**	• Probiotic Therapy • Antibacterial Therapy
**Stem Cell Therapy**	• PDLSCs • GMSCs • Other MSCs
**Gene Therapy**	• PT Associated with PDT
**Other Therapies**	• ICG-diode Laser-based PTT • MB-PDT • Single-photon Treatment • LIPUS

**Table 2:** Destructive matrix metalloproteinases (MMPs) in periodontitis.

Enzyme	Primary Cellular Source	Description
MMP—8	Polymorphonuclear leucocyte (PMN)	Collagenase. A dominant MMP in gingival crevicular fluid in periodontitis
MMP—9	PMN	Gelatinase. Also dominant in gingival crevicular fluid
MMP—13	Bone and epithelium	Collagenase. Dominant MMP in diseased gingival tissues. Mediates pathological bone loss

## Data Availability

The original contributions presented in this study are included in the article Further inquiries can be directed to the corresponding author.
